# Chemical resistance of the gram-negative bacteria to different sanitizers in a water purification system

**DOI:** 10.1186/1471-2334-6-131

**Published:** 2006-08-16

**Authors:** Priscila G Mazzola, Alzira MS Martins, Thereza CV Penna

**Affiliations:** 1 Department of Biochemical and Pharmaceutical Technology, School of Pharmaceutical Sciences, University of São Paulo, Avda. Professor Lineu Prestes, 580, Bloco 16, 05508–900, São Paulo, São Paulo, Brazil

## Abstract

**Background:**

Purified water for pharmaceutical purposes must be free of microbial contamination and pyrogens. Even with the additional sanitary and disinfecting treatments applied to the system (sequential operational stages), *Pseudomonas aeruginosa, Pseudomonas fluorescens, Pseudomonas alcaligenes, Pseudomonas picketti, Flavobacterium aureum, Acinetobacter lowffi and Pseudomonas diminuta *were isolated and identified from a thirteen-stage purification system. To evaluate the efficacy of the chemical agents used in the disinfecting process along with those used to adjust chemical characteristics of the system, over the identified bacteria, the kinetic parameter of killing time (D-value) necessary to inactivate 90% of the initial bioburden (decimal reduction time) was experimentally determined.

**Methods:**

*Pseudomonas aeruginosa, Pseudomonas fluorescens, Pseudomonas alcaligenes*, *Pseudomonas picketti*, *Flavobacterium aureum*, *Acinetobacter lowffi *and *Pseudomonas diminuta *were called in house (wild) bacteria. *Pseudomonas diminuta *ATCC 11568, *Pseudomonas alcaligenes *INCQS *, Pseudomonas aeruginosa *ATCC 15442, *Pseudomonas fluorescens *ATCC 3178, *Pseudomonas picketti *ATCC 5031, *Bacillus subtilis *ATCC 937 and *Escherichia coli *ATCC 25922 were used as 'standard' bacteria to evaluate resistance at 25°C against either 0.5% citric acid, 0.5% hydrochloric acid, 70% ethanol, 0.5% sodium bisulfite, 0.4% sodium hydroxide, 0.5% sodium hypochlorite, or a mixture of 2.2% hydrogen peroxide (H_2_O_2_) and 0.45% peracetic acid.

**Results:**

The efficacy of the sanitizers varied with concentration and contact time to reduce decimal logarithmic (log_10_) population (n cycles). To kill 90% of the initial population (or one log_10 _cycle), the necessary time (D-value) was for *P. aeruginosa *into: (i) 0.5% citric acid, D = 3.8 min; (ii) 0.5% hydrochloric acid, D = 6.9 min; (iii) 70% ethanol, D = 9.7 min; (iv) 0.5% sodium bisulfite, D = 5.3 min; (v) 0.4% sodium hydroxide, D = 14.2 min; (vi) 0.5% sodium hypochlorite, D = 7.9 min; (vii) mixture of hydrogen peroxide (2.2%) plus peracetic acid (0.45%), D = 5.5 min.

**Conclusion:**

The contact time of 180 min of the system with the mixture of H_2_O_2_+ peracetic acid, a total theoretical reduction of 6 log_10 _cycles was attained in the water purified storage tank and distribution loop. The contact time between the water purification system (WPS) and the sanitary agents should be reviewed to reach sufficient bioburden reduction (over 6 log_10_).

## Background

Water is one of the major commodities used by the pharmaceutical industry. It may be presented as an excipient, or used for reconstitution of products, during synthesis, during production of finished product or as a cleaning agent for rinsing vessels, equipment, primary packing materials [1]. Purified water is also commonly used in various preparations for pharmaceutical solutions and other applications such as cleaning of semi-critical devices, cleaning facilities and equipment. It is commonly used as the main component in peritoneal dialysis solutions in hospitals, in nutrient solutions (including baby formula) and liquid nutrient solutions prepared in the hospital nursery, for administration to children and debilitated patients.

Different grades of water quality are required depending on pharmaceutical uses. Control of the quality of water, in particular, the microbiological quality, is a major concern and the pharmaceutical industry devotes considerable resource to the development and maintenance of water purification systems [1].

For this reason, every pharmaceutical, chemical and biotechnological plant related to health products must rely on appropriate water purification system, permitting it to meet its particular requirements, especially as to the problems related to storage and internal distribution. This procedure must guarantee supply according to the volume required and pursuant to the demanded quality consumption points.

Potable water may be used in chemical synthesis and in early stages of cleaning pharmaceutical manufacturing equipment unless there are specific technical or quality requirements for higher grades of water. Water for injection is water for the preparation of medicines for parenteral administration when water is used as a vehicle and for dissolving and diluting substances or preparations for parenteral before use (sterilized WFI). Purified water (satisfies the endotoxins test) is used for cleaning of medical devices before sterilization and preparation of medicinal products other than those that require the use of water which is sterile and/or apyrogenic, dialysis solutions are made of purified water, for example.

**Figure 1:**
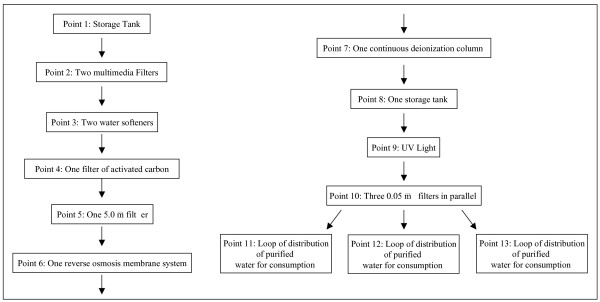
Flow sheet of a typical purification system including the stages and subsequent apparatus from where water points were sampled.

Purified water is obtained from wells and drinking water through a typical water purification system of unit operations presented in a flow sheet in Figure 1, meeting the standards set forth by the 1978/1990 directives issued by the Brazilian Ministry of Health [2].

Water purification systems (WPS) must be validated, according to USP 24 [3] (see Table 1), preventing pyrogen formation. The bacteriological standard ≤ 1.0 Endotoxin Unit (EU/mL) is required for sterile purified water used for washing critical devices before autoclaving.

**Table 1:** USP Standards to purified water and water for injection (WFI)

	Purified Water	WFI
Conductivity	< 1.3 μS/cm (25°C)	< 1.3 μS/cm (25°C)
Total Organic Concentration	< 0.5 ppm	< 0.5 ppm
Bacteria	100 CFU/mL	10 CFU/mL
Endotoxin	Non specified	< 0.25 EU/mL

The WPS that produces, stores and circulates water under background conditions is susceptible to the establishment of adhesive biofilms or microorganisms, which can be the source of undesirable levels of viable microorganisms or endotoxins in the effluent water.

Recent studies have shown that nearly all-large water purification systems can cause biofilm to form in the piping. Biofilm is defined as a microbial community, frequently enclosed in exocellular polymers, that adheres to a surface. They develop on wet surfaces of rooms, equipment and machinery that handle organic matter in non-aseptic conditions (e.g. in pharmaceutical, cosmetic, or food factories, hospitals, kitchens, water pipes, ventilation ducts, etc.) [3].

Biofilms can spread microorganisms within the system and contribute to an increase in particles, in bacteria, and to an increase in the level of total organic carbon (TOC). Contamination can affect the whole process in the pharmaceutical industry or hospital environment. These systems require frequent disinfecting program and microbiological monitoring to ensure water of appropriate microbiological quality (microbial limit at the points of use) (USP 28) [4].

For gram-negative fermenting bacteria in drinking water, the standards show that total coliforms must be less than one colony-forming unit per 100 mL of drinking water. Neither the Brazilian Federal [2] standards nor the USP 28 [4] include levels for gram-negative non-fermenting bacteria, such as the *Pseudomonas *species, which are among the main constituents of biofilms and enterotoxins in purified water [5].

The aim of this research is to analyze the resistance of microorganisms collected from the WPS (from now on called microorganisms in house). The disinfection regime currently used in the water purification system was tested and highlighted in the microbial control of these systems. The resistance was then compared to the standardized microorganisms.

## Methods

In the previous manuscript [6] microorganisms were isolated and identified from thirteen points of a typical water purification system (Fig. 1). The identified microorganisms were: *P. aeruginosa, P. fluorescens*, *P. alcaligenes*, *P. picketti*, *F. aureum*, *A. lowffi *and *P. diminuta*. The standard strains were: *P. diminuta *ATCC 11568, *P. alcaligenes *INCQS *, P. aeruginosa *ATCC 15442, *P. fluorescens *ATCC 3178, *P. picketti *ATCC 5031, *B. subtilis *ATCC 9372 and *E. coli *ATCC 25922. The vegetative strains were maintained on an inclined surface of tryptic soy agar (TSA, Difco, USA) at 4°C, with monthly transfers. The 24 hour cultures grown on TSA at 30–35°C were harvested from tryptic soy broth (TSB, Difco, USA) centrifuged (1000 g/15 min/4°C) and resuspended in saline (0.95 g/mL NaCl plus 0.1 g/mL peptone) to a final population (by pour plate) of 10^6^CFU/mL (colony forming units/mL). These suspensions were used for the D-value tests [6,7]. From each TSA culture, the colonies were transferred to the surface of Cetrimide Agar Base (Difco) in plates and incubated at 30–35°C for 18–24 h. The identification tests used for microorganisms have been previously described [6].

From a stock *B. subtilis *ATCC 9372 suspension, 1 mL was sampled and transferred to 99 mL of sterile saline solution (0.9% NaCl), for dilution purposes (dilution rate 1:100), and kept under magnetic agitation for 15 min, the dilution was repeated (1:100), resulting in a final solution diluted 10^-4^. A 5 mL sample was transferred to a small flask and subjected to thermal shock (80°C/10 min and sudden immersion in a water/ice bath). The initial solution (diluted twice previously, 10^-4^) was then diluted in sterile saline solution to 10^-5^, 10^-6^, 10^-7^, 10^-8^, and 10^-9^, for counting purposes, following 1 mL of each dilution was transferred to a sterile Petri plate, and 8 mL of sterile plate count agar (PCA) was poured in the plate, followed by gentle mixing. The plates were incubated for 24h/35°C) and the number of colonies was counted.

Seven different chemical solutions (disinfectant concentrations appear in the text as w/v) were tested, the reagents were chosen based on which chemical agent (refer to Table 2) is used in each step of the WPS, chemical agents used to pH control and dechlorination were also tested to verify if they caused any loss of viability to the microorganisms.

**Table 2:** Chemical agent, concentration [%], pH values, usage point, contact time (min) and purpose of each solution in the water purification

Chemical Agent	Concentration [%]	pH	Usage Point	Contact Time (minutes)	Purpose
Hydrogen Peroxide + Peracetic Acid	2.2+0.45	2.1	Reverse Osmosis, Deionization	180	Disinfectant
Ethyl Alcohol	70.0	7.2	Sampling Points	1	Disinfectant
Sodium Hypochlorite	0.5	11.9	Storage Tank, Loop of Distribution	60	Disinfectant
Sodium Bissulphate	1.0	4.0	Multimedia Filters, Softener, Carbon Filter	90	Dechlorination
Sodium Hydroxide	0.4	12.8	Reverse Osmosis, Continuous Deionization	30	pH adjustment
Citric Acid	0.5	2.4	Reverse Osmosis	30	pH adjustment
Chloridric Acid	0.5	0.3	Deionization	30	pH adjustment

**Figure 2:**
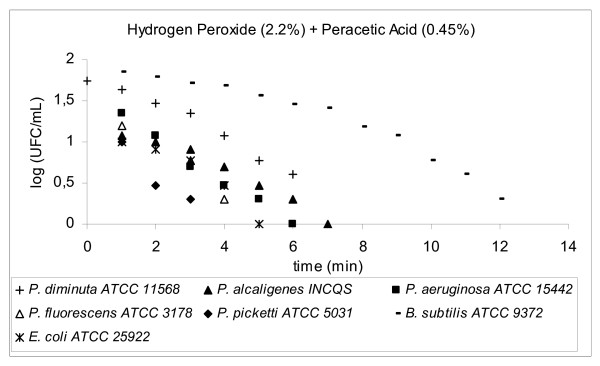
Graphic representation of the standard and in house microorganism reduction against different sanitizers.

**Figure 3:**
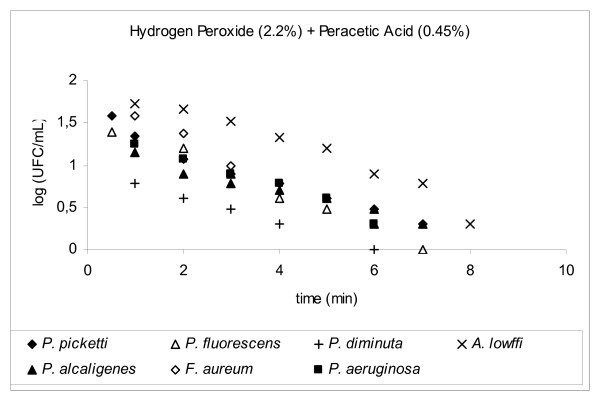
Graphic representation of the standard and in house microorganism reduction against different sanitizers.

**Figure 4:**
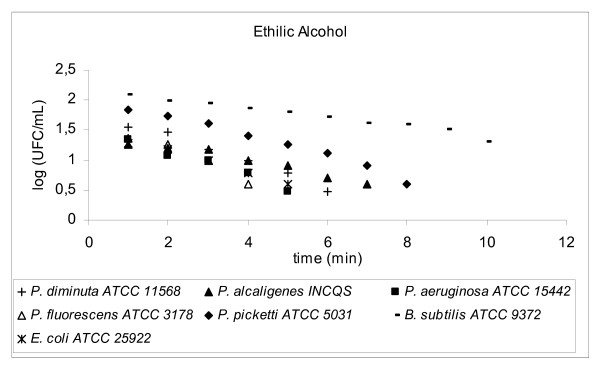
Graphic representation of the standard and in house microorganism reduction against different sanitizers.

**Figure 5:**
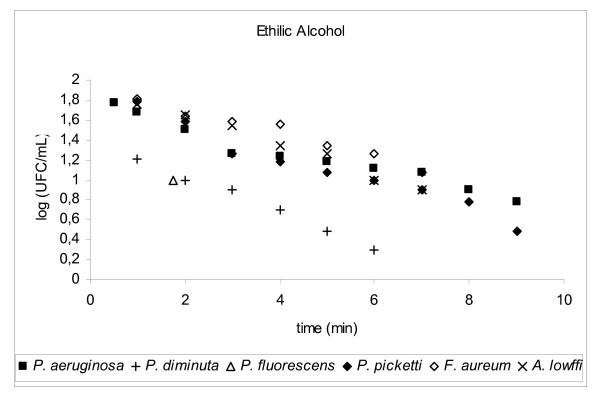
Graphic representation of the standard and in house microorganism reduction against different sanitizers.

**Figure 6:**
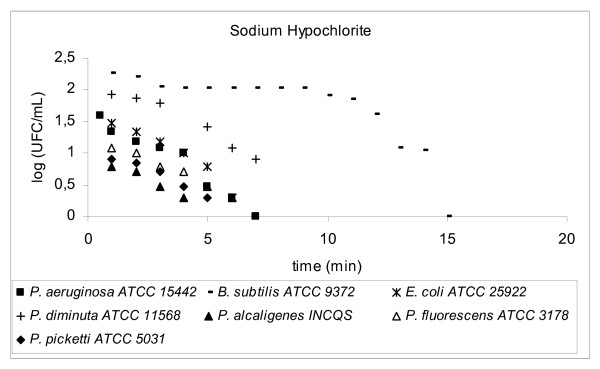
Graphic representation of the standard and in house microorganism reduction against different sanitizers.

**Figure 7:**
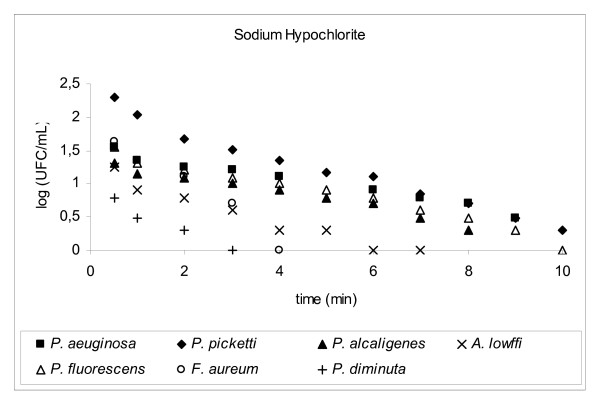
Graphic representation of the standard and in house microorganism reduction against different sanitizers.

Decimal reduction time (D-value) is the interval of time required, under a defined set of conditions, to provide a one decimal logarithm (1 log_10_, n = 1) or 90% reduction in the initial viable bacterial population (bioburden) [8] when exposed to a test disinfectant (chemical agent at final working solution concentration). The determination of D-value involved transferring 1 mL of a 24 h suspension of a standard bacterial strain into 100 mL of a disinfectant solution and kept, with constant agitation, at a controlled temperature (25°C ± 1.0°C). The initial concentration of bacteria (N_o_) exposed to the disinfectant was around 10^5 ^to 10^6 ^CFU/mL, D-values results were plotted *log CFU/mL × time (mins)*, please refer to Figures 2, 3, 4, 5, 6, 7

At regular intervals (1 min for vegetative forms and 5 min for spore forms), 1 mL sample of the mixture (disinfectant solution and microorganism suspension) was transferred to TSB.

For hydrogen peroxide and peracetic acid solution and sodium hypochlorite 1 mL of the inactivating agent – peroxidase 1% and sodium thiosulfate 1%, respectively – was added in 8 mL of TSB to guarantee a complete inactivation of the disinfectant without interfering with survivor growth, the final volume was then 10 mL (8 mL TSB + 1 mL inactivating agent + 1 mL of the mixture microorganism suspension and disinfectant agent).

Ethyl alcohol and other tested solutions were not chemically inactivated, the inactivation was achieved when the sample was in contact with 9 mL of TSB (inactivation by dilution), and the final volume was then 10 mL (9 mL TSB + 1 mL of the mixture microorganism suspension and disinfectant agent).

Using TSA pour plates, the survivors were evaluated by dilution in saline solution (10^4^, 10^3^, 10^2^, 10^1^, 10^0^), it is important to highlight that the initial 30 s of contact between the microorganism strains and the disinfectant solution was enough to reduce 2 log cycles the initial population. A negative control was 9 mL TSB plus 1 mL of an inactivating agent to assure the media sterility. A positive control was made by adding 0.1 mL bacterial suspension into 9.9 mL TSB in a test tube, to guarantee microorganism viability. The assay for each disinfectant and test strain was repeated at least four times. Four samplings of bacterial suspensions for each strain were exposed to the same chemical agent to prepare survivor curves from which the D-values were determined (Fig. 2, 3, 4, 5, 6, 7).

The decimal reduction time (D-value), the interval of time required to reduce one decimal logarithm of the initial bacterial population, at a specified disinfectant concentration (at constant temperature of 25°C), was determined from the negative reciprocal of the slopes of the regression lines, using the linear portions of the survivor curves (log_10 _CFU/mL versus time of exposure to the chemical solution, at constant temperature) [6,7].

The total contact time for microorganism inhibition by the chemical agent was calculated to be equivalent to a 6 log_10 _reduction in viable bioburden to meet the international standard requirements [4,9].

## Results

For a better understanding of a disinfectant's effectiveness and standardization of use in purification system programs, the standard bacterial strains analyzed were established as test microbial suspension. The use of the test microbial suspension is to monitor the disinfection procedure and its performance is dependent on both the initial test microbial suspension population (N_0_) and the D-value [9,10]. The overkill approach to exposure by a disinfectant agent is based on the premise that the extent of treatment will inactivate the initial bioburden (≥ 10^6 ^CFU/mL) and provide an additional safety factor [9,11]. Decimal reduction times (D- values), the number of decimal logarithm reduction (n) for the period of application of every disinfecting solution and bacteria tested. The exposure time for n = 6 reduction for every chemical agent and bacteria are shown in Table 3.

**Table 3:** Decimal reduction times (D- values), and level of confidence (n = number of decimal logarithm reduction) for the period of application of every disinfecting solution and bacteria tested. The exposure time for n = 6 reduction for every chemical agent and bacteria. The table is organized (3.1 –3.7) based on the chemical agent

3.1			
**CITRIC ACID (pH 2.4)**(0.5%, 30 min)	^1^**D-value [minutes]**	^2^**n = 6 log**_10 _**[minutes]**	^3^**n = t/D**

**Microorganism**			
*A. lowffi*	1.77	10.62	16.96
*F. aureum*	15.77	94.64	1.90
*P. aeruginosa*	3.81	22.84	7.88
*P. alcaligenes*	2.99	17.96	10.02
*P. diminuta*	5.43	32.61	5.52
*P. fluorescens*	17.06	102.39	1.76
*P. picketti*	3.29	19.76	9.11
*B. subtilis *ATCC 9372	9.51	57.09	3.15
*E. coli *ATCC 25922	7.73	46.37	3.88
*P. aeruginosa *ATCC 15442	8.95	53.72	3.35
*P. alcaligenes *INCQS	4.54	27.22	6.61
*P. diminuta *ATCC 11568	9.50	56.98	3.16
*P. fluorescences *ATCC 3178	5.32	31.91	5.64
*P. picketti *ATCC 5031	4.35	26.08	6.90

3.2			

**HYDROCHLORIC ACID (pH 0.3)**(5.0%, 30 min)	^1^**D-value [minutes]**	^2^**n = 6 log**_10 _**[minutes]**	^3^**t = n × D**

**Microorganism**			
*A. lowffi*	7.26	43.57	4.13
*F. aureum*	5.67	34.03	5.29
*P. aeruginosa*	6.88	41.29	4.36
*P. alcaligenes*	5.56	33.39	5.39
*P. diminuta*	6.49	38.91	4.63
*P. fluorescens*	9.12	54.74	3.29
*P. picketti*	10.81	64.86	2.78
*B. subtilis *ATCC 9372	10.88	65.29	2.76
*E. coli *ATCC 25922	3.70	22.19	8.11
*P. aeruginosa *ATCC 15442	6.35	38.12	4.72
*P. alcaligenes *INCQS	4.03	24.16	7.45
*P. diminuta *ATCC 11568	2.81	16.87	10.67
*P. fluorescences *ATCC 3178	4.22	25.34	7.10
*P. picketti *ATCC 5031	4.78	28.71	6.27

3.3			

**ETHYL ALCOHOL (pH 7.2)**(70.0%, 1 min)	^1^**D-value [minutes]**	^2^**n = 6 log**_10 _**[minutes]**	^3^**t = n × D**

**Microorganism**			
*A. lowffi*	6.84	41.07	6.84
*F. aureum*	6.05	36.30	6.05
*P. aeruginosa*	9.71	58.25	9.71
*P. alcaligenes*	5.92	35.50	5.92
*P. diminuta*	5.56	33.39	5.56
*P. fluorescens*	6.79	40.73	6.79
*P. picketti*	7.04	42.25	7.04
*B. subtilis *ATCC 9372	9.98	59.88	9.98
*E. coli *ATCC 25922	4.49	26.93	4.49
*P. aeruginosa *ATCC 15442	4.92	29.53	4.92
*P. alcaligenes *INCQS	8.64	51.81	8.64
*P. diminuta *ATCC 11568	4.59	27.55	4.59
*P. fluorescences *ATCC 3178	2.74	16.47	2.74
*P. picketti *ATCC 5031	5.03	30.15	5.03

3.4			

**SODIUM BISULPHITE (pH 4.0)**(0.5%, 90 min)	^1^**D-value [minutes]**	^2^**n = 6 log**_10 _**[minutes]**	^3^**t = n × D**

**Microorganism**			
*A. lowffi*	4.82	28.92	18.68
*F. aureum*	3.70	22.21	24.32
*P. aeruginosa*	5.25	31.53	17.13
*P. alcaligenes*	3.50	21.00	25.71
*P. diminuta*	4.01	24.04	22.46
*P. fluorescens*	6.33	38.00	14.21
*P. picketti*	7.86	47.13	11.46
*B. subtilis *ATCC 9372	9.47	56.82	9.50
*E. coli *ATCC 25922	6.05	36.30	14.88
*P. aeruginosa *ATCC 15442	7.34	44.02	12.27
*P. alcaligenes *INCQS	6.93	41.55	13.00
*P. diminuta *ATCC 11568	6.72	40.32	13.39
*P. fluorescences *ATCC 3178	6.12	36.72	14.71
*P. picketti *ATCC 5031	4.88	29.25	18.46

3.5			

**SODIUM HYDROXIDE (pH 12.8)**(0.4%, 30 min)	^1^**D-value [minutes]**	^2^**n = 6 log**_10 _**[minutes]**	^3^**t = n × D**

**Microorganism**			
*A. lowffi*	12.21	73.26	2.46
*F. aureum*	16.23	97.40	1.85
*P. aeruginosa*	14.16	84.99	2.12
*P. alcaligenes*	11.53	69.20	2.60
*P. diminuta*	18.45	110.70	1.63
*P. fluorescens*	10.96	65.79	2.74
*P. picketti*	16.72	100.33	1.79
*B. subtilis *ATCC 9372	10.96	65.79	2.74
*E. coli *ATCC 25922	5.16	30.94	5.82
*P. aeruginosa *ATCC 15442	5.93	35.57	5.06
*P. alcaligenes *INCQS	4.28	25.67	7.01
*P. diminuta *ATCC 11568	5.60	33.59	5.36
*P. fluorescences *ATCC 3178	4.50	27.00	6.67
*P. picketti *ATCC 5031	4.22	25.30	7.12

3.6			

**SODIUM HYPOCHLORITE (pH 11.1)**(0.5%, 60 min)	^1^**D-value [minutes]**	^2^**n = 6 log**_10 _**[minutes]**	^3^**t = n × D**

**Microorganism**			
*A. lowffi*	5.14	30.85	11.67
*F. aureum*	3.33	19.99	18.01
*P. aeruginosa*	7.91	47.43	7.59
*P. alcaligenes*	8.58	51.50	6.99
*P. diminuta*	3.98	23.89	15.07
*P. fluorescens*	7.29	43.76	8.23
*P. picketti*	5.30	31.80	11.32
*B. subtilis *ATCC 9372	9.32	55.92	6.44
*E. coli *ATCC 25922	4.52	27.10	13.28
*P. aeruginosa *ATCC 15442	4.54	27.21	13.23
*P. alcaligenes *INCQS	6.54	39.27	9.17
*P. diminuta *ATCC 11568	6.43	38.56	9.34
*P. fluorescences *ATCC 3178	6.32	37.93	9.49
*P. picketti *ATCC 5031	6.36	38.17	9.43

3.7			

**HYDROGEN PEROXIDE + PERACETIC ACID (pH 2.3)**(2.2% + 0.45%, 180 min)	^1^**D-value [minutes]**	^2^**n = 6 log**_10 _**[minutes]**	^3^**t = n × D**


**Microorganism**			
*A. lowffi*	4.19	25.15	42.95
*F. aureum*	4.64	27.82	38.83
*P. aeruginosa*	5.53	33.19	32.54
*P. alcaligenes*	4.87	29.23	36.95
*P. diminuta*	5.39	32.31	33.43
*P. fluorescens*	4.36	26.14	41.31
*P. picketti*	5.44	32.63	33.10
*B. subtilis *ATCC 9372	7.44	44.64	24.19
*E. coli *ATCC 25922	4.12	24.73	43.67
*P. aeruginosa *ATCC 15442	3.78	22.66	47.66
*P. alcaligenes *INCQS	6.61	39.66	27.23
*P. diminuta *ATCC 11568	5.39	32.31	33.43
*P. fluorescences *ATCC 3178	3.41	20.47	52.76
*P. picketti *ATCC 5031	2.86	17.17	62.91

^1^D-value = decimal reduction time; (-1/D) = slope.

^2 ^t = n × D and n = 6 log_10;_

t = the exposure time for a 6 log_10 _reduction in the bioburden (No) with a defined D-value

^3^t = n × D, where: t = total exposure time currently used (min); D = D-value determined (min)

In table 3 the decimal reduction times (D-values, min) are presented for the in house and standard strains, respectively, in contact to the chemical agent. The total contact time (t, min) is a multiple of the D-value (min), considering the following relation t = n*D, where *n *is the number of decimal logarithmic reduction in the initial population (log N_0_) of the microorganism, after contact with the chemical agent.

Citric acid (0.5%) when applied to in house strains during 30 min/25°C was able to theoretically (predicted based on the average D-value) reduce 15 log_10 _cycles of *A. lowffi *(D = 1.77 min), the most sensitive bacteria, and reduce theoretically 10 log_10 _cycles of *P. alcaligenes *(D = 2.99 min), they were not supposed to survive the sanitation procedure of the system. This contact time was enough to reduce (5–8 log_10 _cycles) the following microorganism populations: *P. aeruginosa*, *P. picketti*, *P. alcaligenes *INCQS, *P. fluorescens *ATCC 3178, *P. picketti *ATCC 5031. However, *F. aureum*, *P. diminuta *and *P. fluorescencens *showed resistance to the contact with citric acid similar to *B. subtillis *ATCC 9372 (reduction of 2–3 log_10 _cycles). Although citric acid is effective against some of the tested strains (gram-negatives), after 30 minutes of contact it is still possible for these microorganisms to survive in the system. To be effective as a sanitizer the suggested contact time would be 3h30 min to achieve n = 6 log_10_. Citric acid is also used with heated water (100 – 105°C) for 20 hours in dialyser reprocessing, in these conditions all infective agents including spores are destroyed and depyrogenation may occur, however these temperatures may result in structural damage, limiting the use [12]. Citric acid is used for cleaning and adjustment of reverse osmosis pH membrane.

Hydrochloric acid is used for cleaning and adjustment of pH on continuous de-ionization of the unit. When in contact to hydrochloric acid (0.5%) the more resistant strains were *P. picketti *and *B. subtilis *ATCC 9372, both showing reductions lower than n = 3 log_10 _cycles, this is a result that should be highlighted, considering that a wild strain is as resistant as spores of *B. subtilis *ATCC 9372, considered standard strain in high level disinfection procedures [9]. The most sensitive strains were *E. coli *ATCC 25922 and *P. diminuta *ATCC 11568 (n≈3).

Alcohol is used to clean the outer surface of sampling points. Alcohols exhibit rapid broad-spectrum antimicrobial activity against vegetative bacteria (including mycobacteria), viruses, and fungi but are not sporicidal. They are, however, known to inhibit sporulation and spore germination [13], but this effect is reversible [14,15]. Because of the lack of sporicidal activity, alcohols are not recommended for sterilization but are widely used for both hard-surface disinfection and skin antisepsis [16].

Considering the ethanol contact time of 1 minute, the reduction achieved for the tested strains were not enough to reduce the initial population. D-values, were all higher than 1 minute, the lowest being 2.74 min (*P. fluorescens *ATCC3178) therefore the contact time should be at least 16.44 min to avoid sampling cross contamination.

For in house (wild) strains, the sodium bisulphite (0.5%) was able to reduce theoretically more than 13 cycles in 90 minutes (recommended contact time), these strains are *P. aeruginosa *(n = 14); *P. diminuta *(n = 23); *P. fluorescens *(n = 13); *P. alcaligenes *(n = 25); *P. picketti *(n = 21); *F. aureum *(n = 24) and *A lowffi *(n = 18). While on standard strains, *P. diminuta *ATCC 11568 (n = 13); *P. alcaligenes *INCQS (n = 16), *P.aeruginosa *ATCC 15442 (n = 12); *P. aeruginosa *ATCC 27853 (n = 23); *P. fluorescens *ATCC 3178 (n = 13); *P. picketti *ATCC 5031 (n = 21); *B. subtilis *ATCC 9372 (n = 9); *B. subtilis *ATCC 6633 (n = 7), and *E. coli *ATCC 25922 (n = 18). Even though sodium bisulphite is used to preserve and de-chlorine multi-medium filters, softeners and coal filters, it effectively promoted safe level of confidence (n>6) related to the standard and even the wild bacteria isolated from the purified water system, which were not supposed to be found after the disinfection procedure.

Hypochlorites are widely used in healthcare facilities in a variety of settings [17]. Inorganic chlorine solution is also used for disinfecting of counter tops and floors. Hypochlorites are the most widely used of the chlorine disinfectants and are available in a liquid (e.g., sodium hypochlorite) or solid (e.g., calcium hypochlorite) form. They have a broad spectrum of antimicrobial activity (i.e., bactericidal, virucidal, fungicidal, mycobactericidal, sporicidal), do not leave toxic residues, are unaffected by water hardness, are inexpensive and fast acting, [17] remove dried or fixed organisms and biofilms from surfaces, [18] and a low incidence of serious toxicity.

*P. aeruginosa *(n = 18), *P. diminuta *(n = 15), *P. picketti *(n = 13), *E. coli *ATCC 25922 (n = 11), *P. aeruginosa *ATCC15442 (n = 13) were more sensitive to the presence of sodium hypochlorite (0.5%) for 60 minutes. Other tested microorganisms decreased between 6–9 log_10 _cycles, after the contact time. Overall, sodium hypochlorite solution was very effective against the tested stains, keeping a safe level of confidence (n = 6), although it is just used to clean the feeding water tank, the purified water storage tank and distribution loop points.

Sodium hydroxide (0.4%) is used for disinfecting and pH adjustment in reverse osmosis membrane and continuous de-ionization. This solution was able to reduce just 2–3 log_10 _cycles in 30 minutes of all wild strains and *B. subtilis *ATCC 9372. However, the initial population of standard strains was reduced more than 5 log_10 _cycles in 30 minutes.

Minncare™ is used for hygienization of reverse osmosis membranes and continuous de-ionization unit. The association of hydrogen peroxide (2.2%) + peracetic acid (0.45%), Minncare™, was the most effective tested solution against the bacteria strains tested, promoting between 24 and 63 log_10 _reduction in the initial population of *B. subtilis *ATCC 9372 (the most resistant strain), and *P. picketti *ATCC 5031 (the most sensitive strain), respectively.

Hydrogen peroxide (H_2_O_2_) is a widely used biocide for disinfection, sterilization, and antisepsis. It is a clear, colorless liquid that is commercially available in a variety of concentrations ranging from 3 to 90%. H_2_O_2 _is considered environmentally friendly, because it can rapidly degrade into the innocuous products water and oxygen. Although pure solutions are generally stable, most contain stabilizers to prevent decomposition. H_2_O_2 _demonstrates broad-spectrum efficacy against viruses, bacteria, yeasts, and bacterial spores [19]. In general, greater activity is seen against gram-positive than gram-negative bacteria; however, the presence of catalase or other peroxidases in these organisms can increase tolerance in the presence of lower concentrations. Higher concentrations of H_2_O_2 _(10 to 30%) and longer contact times are required for sporicidal activity [20]. Peracetic acid (CH_3_COOOH) is considered a more potent biocide than hydrogen peroxide, being sporicidal, bactericidal, virucidal, and fungicidal at low concentrations (0.3%) [19]. PAA also decomposes to safe by-products (acetic acid and oxygen) but has the added advantages of being free from decomposition by peroxidases, unlike H_2_O_2_, and remaining active in the presence of organic loads [15]. Its main application is as a low-temperature liquid sterilant for medical devices, flexible scopes, and hemodialyzers, but it is also used as an environmental surface sterilant. Similar to H_2_O_2_, PAA probably denatures proteins and enzymes and increases cell wall permeability by disrupting sulfhydryl and sulfur bonds [15,19]

## Discussion

*B. subtilis *ATCC 9372 is considered standard strain in disinfection processes, to assure the confidence level above 6log_10 _of the vegetative bacteria. This strain showed higher resistance than the other tested strains against hydrochloric acid, ethyl alcohol, sodium bisulphate, sodium hypochlorite and Minncare™.

However, *B. subtilis *ATCC 9372 presented similar D-value than: *P. picketti *against citric acid; *P. aeruginosa *and *P. alcaligenes *against hydrochloric acid; *P. aeruginosa *and *P. picketti *against sodium bisulphate; *P. diminuta *and *F. aureum *against sodium hydroxide; *P. alcaligenes *and *P. fluorescencens *against sodium hypochlorite; *P. alcaligenes *against Minncare™. Therefore *F. aureum*, *P. fluorescencens*, presented D-value 1.5 times higher than the D-value observed for *B. subtilis *against citric acid. The wild isolated strains showed up to twice the decimal reduction time than B. subtilis ATCC 9372 against sodium hydroxide, confirming that the evaluation of the efficacy of any chemical disinfectant applied to the disinfection of the WPS should be based on the Gram-negative bacteria isolated from the same system.

Preventive actions should be taken periodically against the spread of microorganisms in the water used in health center areas and in pharmaceutical industries these analysis allow improvements in the WPS rapidly, as required. *Pseudomonas *species and other gram-negative bacteria form sludge (biofilm) which resists cleaning and disinfection procedures and it is a source of pyrogens, these can be avoided if purified water is analyzed [21].

Therefore, the washing of (storage tanks) reservoirs and the sanitizing of distribution circuits should be carried out by determining an established schedule for quality control (bacteriological and chemical) of water systems in risky areas. In this context, the following epidemiological data must be investigated and quantified, principally for industrialized parenteral solutions [22].

Coliform and other fecal indicators must be supplemented by additional indicators to compensate for their inefficiency in monitoring the varied pollution levels. *E. coli *ATCC 25922, utilized as gram-negative test organism of disinfecting procedures, was observed to present lower decimal reduction time than the wild gram-negative isolated strains against the majority of the disinfecting agents assayed. This additional procedure could prove to be adequate for identification of several other groups of microorganisms, to wit: heterotrophic bacteria, virus, yeast, *Pseudomonas aeruginosa *and *Staphylococcus aureus*.

## Conclusion

Microorganisms isolated from the water purification system showed a higher resistance to chemical disinfecting agents than the standard strains tested. One possible reason is the widespread use of biocides, as used in water supplies and water treatment systems, act to provide continuous selection pressure.

As many surfaces in the WPS can harbor microorganisms, periodic analysis of treated water is mandatory to prevent biofilm formation and the spread of microorganisms in the system. This work emphasized the removal of gram-negative non-fermenting bacteria, which exhibited a greater resistance to the chemical agents commonly used in the system.

The contact time between the WPS and the sanitary agents should be reviewed to reach sufficient bioburden reduction (over 6 log_10_). Some measures such as washing storage tanks deionization columns, reverse osmosis membrane, as well the sanitation of distribution circuits should be established for quality control (biological and chemical) of water systems.

Water purification system re-disinfecting will be performed in order to verify the microorganism resistance variation after this process. It is important to analyze the initial microorganism population in the system in each one of the thirteen points, and assure its concentration is not greater than 10^2^CFU/mL [5], especially before the reverse osmosis to avoid membrane injuries, increasing the maintenance costs of the process.

## Competing interests

The author(s) declare that they have no competing interests.

## Authors' contributions

PGM carried out D-value experiments and literature review. AMSM carried out microorganism identification and culture growth, also helped on D-value determination. TCVP conceived the research, and participated in its design and coordination. All authors read and approved the final manuscript file.

## Pre-publication history

The pre-publication history for this paper can be accessed here:


